# Genotypic Variation in Photosynthesis and Biomass Partitioning Underlies Agronomic Performance and Cannabinoid Profile in *Cannabis sativa* Under Drought

**DOI:** 10.3390/plants14243840

**Published:** 2025-12-17

**Authors:** Mateus M. Pena, Felipe R. Miranda, Thiago O. Ribeiro, Gustavo C. S. Couto, Sérgio B. F. Rocha, Samuel C. V. Martins, Fábio M. DaMatta

**Affiliations:** 1 National Institute of Science and Technology on Plant Physiology Under Stress Conditions, Departamento de Biologia Vegetal, Universidade Federal de Viçosa, Viçosa 36570-900, MG, Brazil; mateus.pena@ufv.br (M.M.P.); felprm@gmail.com (F.R.M.); thiago.o.ribeiro@ufv.br (T.O.R.); gustavo.couto@ufv.br (G.C.S.C.); samuel.martins@ufv.br (S.C.V.M.); 2 Departamento de Agronomia, Universidade Federal de Viçosa, Viçosa 36570-900, MG, Brazil; sergio.rocha@ufv.br

**Keywords:** carbon allocation, cannabinoid yield, drought stress, harvest index, photosynthetic performance

## Abstract

Drought is a major constraint on *Cannabis sativa* productivity and cannabinoid yield, yet the physiological mechanisms underlying genotypic variation in drought responses remain poorly understood. We hypothesized that (i) more vigorous genotypes would sustain higher photosynthetic rates, (ii) drought would constrain photosynthesis through both diffusional and non-diffusional limitations, and (iii) water deficits would alter cannabinoid production in a genotype-dependent manner. To test these hypotheses, two contrasting genotypes (one tetrahydrocannabinol- (THC) dominant and another cannabidiol- (CBD) dominant) were grown under greenhouse conditions, with water deficit imposed at early flowering. Water deficit induced neither osmotic nor elastic adjustment in either genotype. Although CBD plants accumulated more biomass, they did not exhibit higher photosynthetic rates under well-watered conditions. Under drought, THC plants relied primarily on stomatal regulation, whereas CBD plants showed additional nonstomatal impairments, resulting in stronger declines in photosynthesis. Despite contrasting photoprotective adjustments, both genotypes converged to similar oxidative damage, suggesting that photoprotection was not decisive for their physiological divergence. At the agronomic level, THC plants maintained a higher harvest index under drought, greater baseline cannabinoid concentrations, and inflorescence biomass with higher energetic value. In CBD plants, drought-induced reductions in cannabinoid content and harvest index largely reflected greater photosynthetic impairment and less efficient carbon use. Overall, the resilience of *C. sativa* to drought imposed at early flowering appears to depend less on hydraulic stability and more on sustaining photosynthetic performance, secondary metabolism, and efficient biomass partitioning. These traits represent key targets for breeding genotypes better adapted to cultivation under increasingly variable water availability.

## 1. Introduction

Among abiotic stresses, drought is a major constraint to plant performance, limiting growth and productivity and often reshaping resource allocation in crops [1,2]. The magnitude of its effects depends on the intensity and duration of stress, as well as on genotypic traits and plant developmental stage [3]. One of the earliest physiological responses to declining soil water availability is the inhibition of cell expansion, directly caused by reduced turgor as tissues lose water [4,5]. This response usually leads to reductions in leaf area and plant stature; however, biomass may also be preferentially allocated to roots, thereby enhancing soil exploration and water uptake capacity [6]. In addition, plants often adjust their water relations through osmotic adjustment—via the accumulation of compatible solutes to sustain turgor—and elastic adjustment, involving changes in cell wall mechanical properties that moderate the decline in water potential (Ψ_w_) with water loss. Such adjustments help to establish favorable Ψ_w_ gradients that facilitate water uptake and/or retention, thereby contributing to homeostasis under drought and mitigating its negative impacts [7].

To conserve water, plants typically partially close stomata under drought [6], leading to reduced stomatal conductance (*g*_s_). As a result, both transpiration and CO_2_ diffusion into the leaf are restricted, ultimately limiting photosynthesis [3,6]. In parallel, mesophyll conductance (*g*_m_), which governs CO_2_ diffusion to the chloroplast stroma, also tends to decrease, further reinforcing diffusional limitations to photosynthesis [5,8]. As drought intensifies, photosynthesis is additionally constrained by photochemical limitations, including reduced ATP and NADPH production, impairing RuBP regeneration, and by biochemical constraints such as decreased RuBisCO activity [5,9]. Thus, photosynthesis under drought reflects the interplay of diffusional and non-diffusional limitations, whose relative importance depends on stress intensity and duration, genotype, atmospheric evaporative demand, and previous drought exposure [5,10]. In any case, as photosynthesis declines, part of the absorbed light energy cannot be dissipated through photochemical processes, favoring the overproduction of reactive oxygen species (ROS). Although enzymatic (e.g., superoxide dismutase, SOD; catalase, CAT; peroxidases, POX) and non-enzymatic (e.g., ascorbate, glutathione, carotenoids, phenolics, proline) antioxidant systems act to scavenge ROS, insufficient antioxidant responses may lead to oxidative damage, including lipid peroxidation, pigment degradation, and impaired chloroplast membranes and proteins [11,12].

*Cannabis sativa* L., an annual and predominantly dioecious species, has gained increasing agronomic and biomedical interest owing to its production of fibers, seeds, and recreational and medicinal cannabinoids, particularly Δ^9^-tetrahydrocannabinol (THC) and cannabidiol (CBD) [13]. These compounds accumulate in glandular trichomes, especially on pistillate inflorescences of female plants, which bear higher trichome densities than other tissues [14]. Under drought, cannabis exhibits typical plant responses, including impaired water status accompanied by reductions in *g*_s_ and net photosynthesis rates (*A*) [13]. Recent studies in hemp and medicinal cannabis have documented wide variation in stomatal sensitivity to drought, reflecting adaptive diversity and potential for selecting more resilient genotypes [13,15,16]. Nevertheless, the mechanistic basis of reduced photosynthetic performance under drought in cannabis remains only partially understood and warrants further investigation.

From a metabolic and productive perspective, drought can decrease biomass accumulation and floral yield in cannabis, but its effects on cannabinoid content are variable and depend on stress intensity, developmental stage, and genotype [13]. Controlled studies suggest that moderate, late-stage drought (during advanced flowering) may increase THC and CBD concentrations without penalizing inflorescence mass, whereas severe or early drought tends to reduce both yield and cannabinoid levels [17,18,19]. Overall, the available evidence points to nonlinear, genotype-specific responses.

Because cannabis cultivation requires relatively few agricultural inputs, the species may be suitable for production under reduced water availability, even when yield is partially compromised, as reported for industrial hemp [20]. Understanding how cannabis genotypes respond physiologically and productively to water deficit is therefore critical, particularly in light of the projected increase in drought frequency and severity under climate change [21]. Despite recent advances, little is known about how genotypes that differ in overall vigor and morphological architecture—rather than in cannabinoid profile per se—adjust their water relations, photosynthetic processes, and biomass allocation under drought. Moreover, the mechanisms through which water deficit limits photosynthesis in cannabis, especially the relative contributions of stomatal and non-stomatal constraints, remain insufficiently resolved. Existing studies rarely integrate these photosynthetic responses with drought-induced shifts in water relations, carbon allocation, and secondary metabolism, offering limited insight into how contrasting genotypes coordinate these processes and how such physiological differences ultimately influence growth and cannabinoid production under water limitation. We hypothesized that (i) more vigorous genotypes, characterized by greater biomass accumulation, would also sustain higher photosynthetic rates; (ii) drought would constrain photosynthesis through both stomatal and nonstomatal limitations, with genotypic differences in their relative contribution; and (iii) water deficit would alter the production of major cannabinoids (CBD and THC) in a genotype-dependent manner, reflecting variation in photosynthetic performance and water status. By comparing two genotypes that differ broadly in growth habit and metabolic profile—but were not selected as explicit chemotype representatives—under controlled water regimes, this study provides a mechanistic assessment of how contrasting cannabis genotypes cope with water deficit by integrating physiological, biochemical, and productive responses.

## 2. Results

Because measurements at 50% and 30% field capacity (FC) were conducted on different days, under potentially varying environmental conditions, the responses observed at these two soil moisture levels are not strictly comparable. Therefore, control plants were evaluated at both time points, and the treatment effects are presented here as comparisons between well-watered and water-stressed plants within each soil moisture level.

To place the results in a broader quantitative and biological context, effect size analysis (partial η^2^; Table S1) revealed a clear hierarchy in the relative importance of the experimental factors across trait categories. Overall, traits related to plant–water relations and gas exchange were largely governed by water availability at both soil moisture levels, underscoring the dominant role of drought in constraining plant function, with only a few parameters (e.g., *A*) showing comparable contributions of water availability and genotype at 30% FC. In contrast, biochemical and metabolic traits exhibited a more nuanced pattern: antioxidant enzyme activities reflected similar contributions of genotype and water availability, whereas oxidative damage, as indicated by malondialdehyde (MDA) accumulation, was predominantly driven by water deficit. Primary carbohydrate metabolism was also mainly controlled by water availability, particularly for hexoses, while genotype exerted a stronger influence on sucrose and starch pools, consistent with inherent differences in carbon allocation strategies. Strikingly, cannabinoid profiles and biomass-related traits were overwhelmingly determined by genotype, highlighting strong genetic control over these economically relevant traits, whereas inflorescence biomass showed comparable contributions of genetic and environmental factors. Overall, genotype × water interaction effects were generally small, indicating that genotypic differences were largely conserved across water regimes and that the main effects of genotype and drought dominated the observed responses. Notable exceptions were detected for major cannabinoid pools and inflorescence biomass, for which interaction effects were more pronounced.

**Figure 1:**
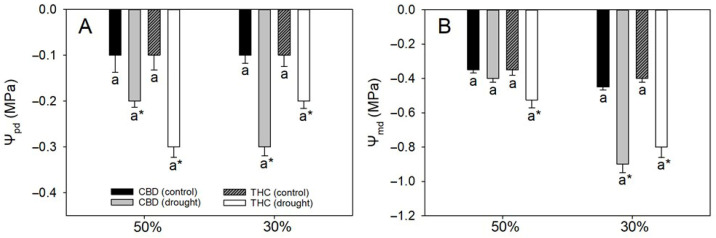
(**A**) Predawn water potential (Ψ_pd_) and (**B**) midday water potential (Ψ_md_) in two cannabis genotypes grown under well-watered or drought conditions. For droughted plants, measurements were taken at 50% and 30% field capacity (FC). Values are means ± SE (*n* = 8). Different letters indicate significant differences between genotypes within the same water regime; asterisks, when shown, indicate a drought effect within a genotype (Tukey’s test, *p* ≤ 0.05).

As expected, both leaf Ψ_w_ at predawn (Ψ_pd_) and midday (Ψ_md_) declined under drought, except for Ψ_md_ in CBD plants under 50% FC (Figure 1). Interestingly, Ψ_pd_ remained relatively stable as soil water content declined from 50% to 30% FC, suggesting a limited capacity of predawn values to reflect progressive stress severity. By contrast, Ψ_md_ exhibited a marked reduction (from −0.4/−0.5 MPa to −0.8 MPa) in both genotypes, highlighting its greater sensitivity to short-term diurnal water deficits. At 30% FC, no genotypic differences were detected in either Ψ_pd_ or Ψ_md_, indicating comparable water status with increasing drought severity (Figure 1).

From pressure–volume curves, the osmotic potential at full turgor (Ψ_s(100)_) and at the turgor loss point (Ψ_s(0)_), and the bulk modulus of elasticity (ε) were essentially similar across genotypes and unaffected by the treatments (Table 1). Leaf capacitance at full turgor (*C*_(100)_) remained stable across treatments, whereas capacitance at the turgor loss point (*C*_(0)_) in THC plants decreased by 50% under drought compared with control values (Table 1).

**Figure 2:**
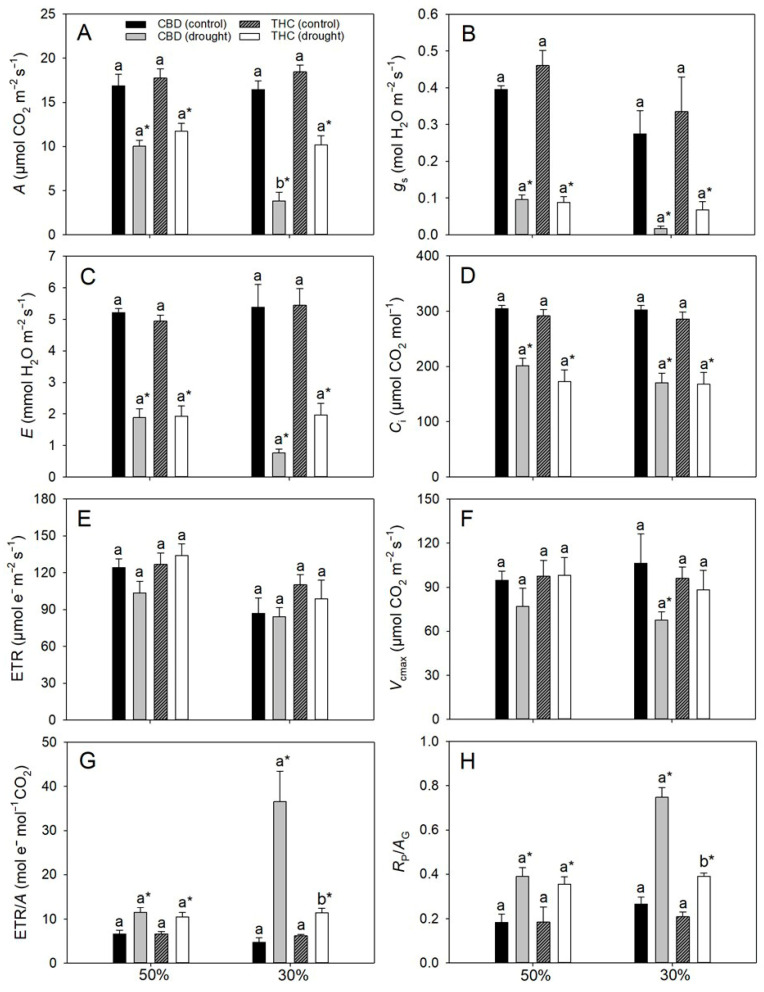
(**A**) Net photosynthetic rate (*A*), (**B**) stomatal conductance (*g*_s_). (**C**) internal CO_2_ concentration (*C*_i_), (**D**) transpiration rate (*E*), (**E**) electron transport rate (ETR), (**F**) maximum single-point carboxylation capacity based on chloroplastic CO_2_ concentration (*V*_cmax_), (**G**) electron transport rate-to-photosynthesis ratio (ETR/*A*), and (**H**) photorespiration-to-gross photosynthesis ratio (*R*_P_/*A*_G_) in two cannabis genotypes grown under well-watered or drought conditions. For droughted plants, measurements were taken at 50% and 30% field capacity (FC). Values are means ± SE (*n* = 8). Different letters indicate significant differences between genotypes within the same water regime; asterisks, when shown, indicate a drought effect within a genotype (Tukey’s test, *p* ≤ 0.05).

Under full irrigation, gas-exchange parameters did not differ between genotypes. In contrast, drought stress markedly altered these traits (Figure 2). At 50% FC, *A* declined by approximately 40%, accompanied by sharp reductions in *g*_s_ (76–81%), intercellular CO_2_ concentration (*C*_i_, 32–48%), and transpiration rate (*E*, ~67%) relative to control plants, irrespective of genotype (Figure 2A–D). Conversely, the electron transport rate (ETR) and the single-point apparent maximum carboxylation capacity of RuBisCO, based on chloroplastic CO_2_ concentration (*V*_cmax_), remained unaffected (Figure 2E,F), whereas the ETR/*A* and photorespiration to gross photosynthesis (*R*_p_/*A*_G_) ratios nearly doubled under drought in both genotypes (Figure 2G,H). At 30% FC, decreases in *A* and *g*_s_ became even more pronounced, particularly in CBD, reaching ~20% and 6%, respectively, of the values observed in irrigated plants (in THC, the corresponding values were 44% and 20%, as can be deduced from Figure 2A,B). In THC, however, *A* did not decline further relative to 50% FC, whereas *E* was strongly reduced (Figure 2D). While ETR remained stable, *V*_cmax_ decreased by 37% exclusively in CBD (Figure 2E,F). Furthermore, both ETR/*A* and *R*_p_/*A*_G_ ratios plateaued at 30% FC in THC plants but in CBD they were 242% and 189% higher, respectively, than in well-watered controls (Figure 2G,H). Regardless of drought severity, *A* and *g*_s_ were strongly and positively correlated (*r* ≥ 0.79, *p* < 0.05) in both genotypes.

We additionally assessed some chlorophyll (Chl) *a* fluorescence (only at 30% FC) and showed similar drought responses in both genotypes (Table 2). In control plants, the maximum quantum efficiency of PSII photochemistry (*F*_v_/*F*_m_) values averaged 0.81–0.83, but declined by approximately 20% under drought, a reduction accompanied by marked increases in the minimum fluorescence (*F*_0_, 52–66%), whereas the coefficient of photochemical quenching (*q*_L_) strongly decreased by 59–73% and the non-photochemical quenching (NPQ) reduced by 36–40%; nevertheless, the absolute values of NPQ were consistently higher in THC (81–98%) than in CBD plants, irrespective of water availability (Table 2).

Antioxidant enzyme activities declined overall under drought (Table 3). SOD activity decreased by 36% in CBD and 50% in THC, while CAT was reduced by 30% and 22%, respectively, in these clones. Despite these reductions, both SOD and CAT activities remained higher in CBD than in THC plants, an advantage that became even more evident under drought, with activities 78% (SOD) and 45% (CAT) greater in CBD. In contrast, POX activity showed a genotype-specific response: it dropped sharply in CBD (58%) but was unaffected in THC, resulting in similar levels between genotypes under drought. Regardless of these enzymatic differences, MDA concentrations increased by ~23–29% in both genotypes, indicating comparable oxidative damage under water deficit (Table 3).

Metabolic analyses revealed pronounced shifts in metabolite profiles under water deficit (Table 4). Total Chl (*a* + *b*) decreased by 33% in CBD and 18% in THC plants, resulting in 32% higher Chl pools in droughted THC individuals. Carotenoid concentrations also declined, by 40% in CBD and 27% in THC. Regardless of genotype, drought induced significant reductions in free amino acids (~30%) and in carbohydrates, as evidenced by decreases in concentrations of glucose (~35%), fructose (~22%), sucrose (~22%), and starch (19% in CBD and 39% in THC). Glucose and fructose concentrations did not differ between genotypes; however, sucrose pools were consistently higher (~15%) in THC than in CBD plants. Conversely, starch concentrations were markedly lower in THC plants—by 33% under irrigation and 42% under drought—relative to CBD. Total phenols were unaffected by drought but remained slightly higher in CBD than in THC. Proline concentration showed no significant genotypic differences under either water regime, although it declined slightly under drought in both cultivars (Table 4).

**Figure 3:**
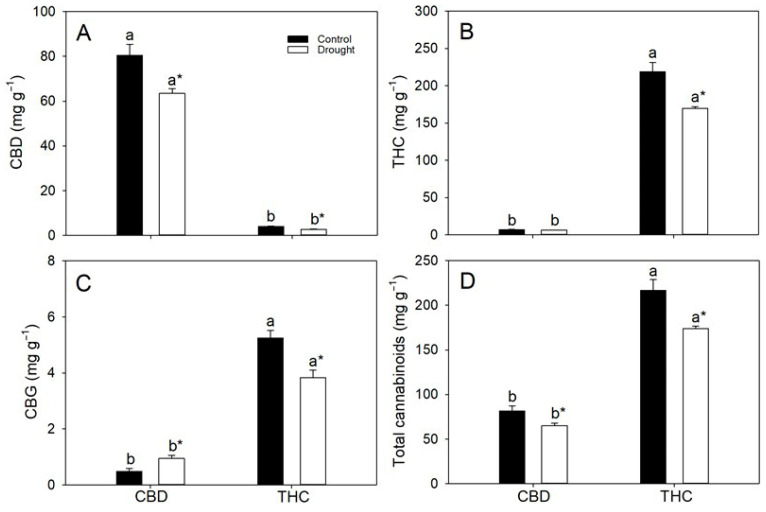
Leaf concentrations (per dry mass) of (**A**) cannabidiol (CBD), (**B**) tetrahydrocannabinol (THC), (**C**) cannabigerol (CBG) and (**D**) total cannabinoids in two cannabis genotypes grown under well-watered or drought conditions. Values are means ± SE (*n* = 8). Different letters indicate significant differences between genotypes within the same water regime; asterisks, when shown, indicate a drought effect within a genotype (Tukey’s test, *p* ≤ 0.05).

As expected, CBD concentrations were substantially higher in the CBD genotype (~7%, *w*/*w*) than in the THC genotype (~0.3–0.4%). Conversely, the THC genotype accumulated markedly greater THC pools (16.8–20.7%) than the CBD genotype (0.63–0.68%) (Figure 3). Water deficit significantly reduced THC and CBD concentrations in both genotypes, decreasing THC levels by 7% and 19% and CBD concentrations by 14% and 25% in the CBD and THC genotypes, respectively (Figure 3A,B). Total cannabigerol (CBG) levels were low (<0.1%) in CBD plants and were increased (96%) by drought, whereas in THC individuals they were consistently higher (~0.4–0.5%) but declined by 24% under water deficit (Figure 3C). Regardless of water regime, THC plants maintained substantially higher total cannabinoid pools—approximately 1.7-fold those of CBD plants (Figure 3D).

**Figure 4:**
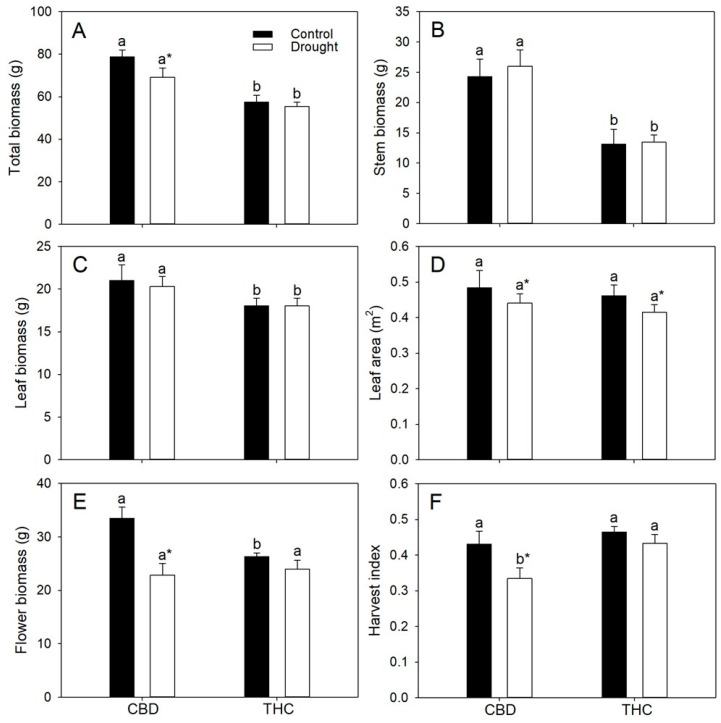
(**A**) Total biomass, (**B**) stem biomass, (**C**) leaf biomass, (**D**) leaf area, (**E**) inflorescence biomass, and (**F**) harvest index (HI) in two *Cannabis* genotypes grown under well-watered or drought conditions. Values are means ± SE (*n* = 8). Different letters indicate significant differences between genotypes within the same water regime; asterisks, when shown, indicate a drought effect within a genotype (Tukey’s test, *p* ≤ 0.05).

Relative to THC, both well-watered and droughted CBD plants accumulated greater total biomass (by 37% and 25%, respectively), primarily due to their higher stem biomass (85% and 94%), whereas leaf biomass was comparable between genotypes (Figure 4A–C). The greater stem mass of CBD plants was mainly associated with their larger stem diameter (~40%), as plant height did not differ between genotypes. Total biomass decreased by 21% in response to water deficit exclusively in CBD (Figure 4A). In contrast, total leaf area did not differ between genotypes but declined by approximately 10% under drought, irrespective of genotype (Figure 4D). Inflorescence biomass was higher in well-watered CBD plants but similar between genotypes under drought; however, drought reduced inflorescence biomass by 32% in CBD plants only (Figure 4E). Consequently, harvest index (HI) decreased by 22% in these plants relative to their well-watered counterparts, although HI values in well-watered CBD plants remained comparable to those observed in THC plants under both water regimes (Figure 4F).

## 3. Discussion

### 3.1. Stable Leaf Water Relations Fail to Explain Divergence in Growth and Photosynthetic Performance

As expected, plants under drought showed reductions in both Ψ_pd_ and Ψ_md_, which became more pronounced as water deficit intensified. Genotypic differences in these parameters were negligible; moreover, there was no evidence of osmotic adjustment (as indicated by the invariance of Ψ_s(100)_ and Ψ_s(0)_) nor of elastic adjustment, since ε also remained constant across water regimes and genotypes [22,23]. Therefore, genotypic differences in drought tolerance regarding photosynthetic performance are unlikely to be attributable to plant–water relations per se. Importantly, Ψ_md_ consistently exceeded Ψ_s(0)_ by ≥0.2 MPa, indicating that leaves did not reach the turgor-loss point; accordingly, the deficits imposed qualify as mild at 50% FC and moderate at 30% FC. Both genotypes exhibited relatively low ε, implying that large changes in Ψ_w_ require substantial water loss and providing a hydraulic buffer that dampens the diurnal gradient between Ψ_pd_ and Ψ_md_ [24]. These features likely explain (i) the modest absolute decreases in Ψ_pd_ and Ψ_md_ across deficit levels and (ii) the relatively small separation between them. Overall, the observed divergence in photosynthetic performance between genotypes is more plausibly attributable to non-hydraulic controls, as detailed below.

Maintenance of water relation parameters such as ε, Ψ_s(100)_ and Ψ_s(0)_ suggests differences in growth ability should not be attributed to varying hydrostatic pressures and/or cell-wall elasticity properties between genotypes. In contrast, hydraulic capacitance revealed a genotype-specific adjustment: while *C*_(100)_ remained unchanged, *C*_(0)_ decreased by 50% in THC plants under drought relative to controls (Table 1). This reduction may indicate a diminished capacity to buffer declines in leaf Ψ_w_ once turgor is lost, potentially exacerbating desiccation stress in this genotype.

### 3.2. Contrasting Stomatal Versus Non-Stomatal Limitations Reveal Divergent Strategies of Photosynthetic Response to Drought

The initial reductions in *A*, observed under 50% FC, were primarily attributable to diffusional limitations at the stomatal level, regardless of genotype. This interpretation is supported by the strong correlation between *A* and *g*_s_, the decline in *C*_i_ —resulting from a proportionally greater reduction in *g*_s_ than in *A* (on average c. 78% and 43%, respectively; Figure 2A,B)—and the maintenance of both photochemical activity (stable ETR) and the intrinsic biochemical capacity for CO_2_ fixation (*V*_cmax_ unchanged). As the water deficit intensified (30% FC), however, the mechanisms limiting photosynthesis diverged between genotypes. In THC, reductions in *A* remained predominantly linked to stomatal closure. In contrast, in the CBD genotype, nonstomatal factors also appeared to contribute substantially to the observed decreases in *A*. This inference is supported by the similar *C*_i_ values between genotypes, despite significantly lower *A* and *V*_cmax_ in CBD plants. Together, these findings indicate distinct drought-response strategies: THC plants rely predominantly on stomatal regulation, thereby preserving the functionality of the photosynthetic machinery under stress, whereas CBD exhibits greater susceptibility to biochemical (and potentially mesophyll) limitations [25]. From an ecophysiological standpoint, these contrasting strategies have important implications for field performance. Genotypes like THC, whose photosynthetic decline is dominated by stomatal limitations, are expected to recover more rapidly following rehydration, an advantageous trait under intermittent drought. Conversely, genotypes such as CBD, in which stress impairs nonstomatal processes, are likely to exhibit slower and often incomplete recovery of photosynthesis, increasing the risk of productivity losses under prolonged or recurrent drought [10].

### 3.3. Failure to Upregulate Photoprotective Pathways Enhances Photosynthesis Photoinhibition and Oxidative Stress During Water Deficit

ETR values, regardless of treatment, were fully sufficient to sustain the measured *A* rates [26], even in the face of drought-induced decreases in Chl concentration. Under drought, however, a marked increase in the ETR/*A* ratio was detected, particularly in CBD plants at 30% FC, where values were more than 200% higher than in THC counterparts. Such an imbalance indicates that the relative electron flux exceeded the capacity for carbon fixation, a condition often associated with electron over-reduction and increased excitation pressure on PSII [27]. In agreement, pronounced declines in *q*_L_ were observed, reflecting reduced openness of PSII reaction centers and a perturbed balance between photochemical excitation and electron utilization in downstream metabolism [28]. This scenario points to enhanced oxidative pressure within chloroplasts, which may ultimately culminate in oxidative stress and photoinhibition [27]. Although part of the excess electron pressure may be alleviated through photorespiration (particularly in CBD plants) [29,30], the lower NPQ values indicate that thermal dissipation of excess energy via the xanthophyll cycle was insufficiently upregulated [31,32]. This interpretation is consistent with the observed decreases in carotenoid pools, further constraining the capacity for non-photochemical quenching. Consequently, plants exhibited clear signs of PSII photoinhibition, as indicated by drought-induced declines in *F*_v_/*F*_m_, suggesting that a fraction of the reaction centers became damaged or temporarily inactivated [33,34]. In support, *F*_0_ values rose markedly even after ≈2 h of dark acclimation. While such increases do not unequivocally confirm chronic photoinhibition, particularly in the absence of overnight dark adaptation, they nonetheless suggest a sustained excitation pressure on PSII and a potential exacerbation of ROS formation. Given that antioxidant enzyme activities were concurrently downregulated under drought, the detoxification of ROS was impaired. Interestingly, despite this general downregulation, CBD plants exhibited relatively higher SOD and CAT activities, along with greater pools of total phenolics, compared with THC. These adjustments, however, were insufficient to counterbalance the greater oxidative burden in CBD. Ultimately, the interplay between excitation pressure and antioxidant defenses converged toward a similar outcome in both genotypes, as reflected by comparable oxidative damage, evidenced by elevated (and similar) MDA levels, a marker for oxidative damage [1].

### 3.4. Genotypic Differences in Photosynthesis, Biomass Partitioning and Cannabinoid Yield Determine Agronomic and Energetic Returns Under Varying Water Availability

No significant reduction in leaf and stem biomass was observed under drought, most likely because water deficit was imposed after the onset of flowering—a stage at which vegetative growth markedly declines or even ceases in annual species [35]. The greater total biomass of CBD plants, however, cannot be attributed to higher *A* per unit leaf area, differences in leaf area, leaf carbohydrate concentrations, or altered cell-wall elastic properties, as previously discussed. Rather, CBD plants invested disproportionately in stem growth, suggesting a tendency to allocate more carbon to structural tissues at the expense of reproductive organs, particularly under stress. In contrast, the stability of HI in THC plants indicates a greater capacity to maintain reproductive partitioning during drought, reflecting enhanced tolerance of both photosynthetic and assimilate allocation processes. Consistent with this, the sharp decrease in starch pools accompanied by a modest rise in sucrose concentration suggests the mobilization of stored carbohydrates to sustain assimilate export toward reproductive sinks, thereby promoting more efficient carbon use under water-limited conditions.

Under well-watered conditions, CBD and THC plants differed in inflorescence biomass but displayed similar HI. However, genotypic differences might emerge if HI were expressed on an energetic basis [36]. Because cannabinoids (CBD, THC, and CBG) are highly reduced terpenoid isomers (C_21_H_30_O_2_) with low oxygen content, their combustion enthalpy (~38 MJ kg^−1^) is comparable to that of lipids and more than twice that of carbohydrates or proteins [37]. Consequently, the markedly lower cannabinoid concentrations in CBD plants (≈40% of those in THC) directly translated into a lower energy density of their inflorescence biomass.

Water deficit imposed at early flowering reduced both CBD and THC concentrations, in agreement with previous studies [18,19], which have shown that drought stress can impair photosynthetic performance and, consequently, cannabinoid biosynthesis [16,38]. In the present study, the pronounced decline in *A* under drought sharply reduced both HI and total cannabinoid concentration in CBD plants. In THC plants, however, the more moderate decrease in *A*—coupled with greater carbohydrate mobilization—was insufficient to compromise inflorescence yield or HI, although energy content declined proportionally to the reduction in cannabinoid concentration. Importantly, the reduction in CBG concentration observed in the THC genotype under drought may indicate functional upregulation, or at least maintenance, of key enzymes in the cannabinoid biosynthetic pathway [18], in sharp contrast to CBD plants, which instead exhibited increased CBG pools under stress. In any case, both genotypes experienced drought-induced decreases in cannabinoid accumulation, underscoring the sensitivity of secondary metabolism to water limitation. Yet, because THC plants consistently accumulated higher baseline levels of cannabinoids, their biomass retained substantially greater energetic value even under drought. In contrast, the combined effects of lower HI and reduced cannabinoid content in CBD plants resulted in biomass with comparatively lower agronomic and energetic returns. Taken together, these findings indicate that genotypic differences in carbon allocation and metabolic plasticity underlie the contrasting agronomic and energetic outcomes observed. The superior capacity of THC plants to maintain reproductive partitioning and preserve high-energy secondary metabolites under drought confers a dual advantage—greater resilience and higher energy yield per unit biomass.

## 4. Materials and Methods

### 4.1. Plant Material, Experimental Design, and Analytical Procedures

The experiment was carried out in Viçosa (20°45′14″ S, 42°52′53″ W; 650 m a.s.l.), southeastern Brazil. Two short-day–adapted cannabis genotypes (clones) were used, differing in growth habit and capacity to produce major cannabinoids: one accumulating predominantly THC with lower biomass (hereafter referred to as THC genotype) and the other accumulating CBD with greater biomass production (CBD genotype). Visual observations indicated that CBD plants displayed more vigorous early growth, characterized by a greater number of smaller leaves compared with THC plants. Importantly, these genotypes were not selected on the basis of drought-related traits, and no *a priori* differences in drought tolerance were expected; their contrasting vigor and metabolic profiles simply provided a convenient framework for examining physiological responses to water deficit.

After successful rooting in a controlled mist chamber, 32 uniform seedlings (16 per genotype) were transplanted into 10 L pots filled with a commercial substrate (Mecplant^®^). Plants were maintained in a greenhouse under naturally fluctuating conditions with temperatures ranging from 18.9 ± 0.8 to 29.7 ± 1.1 °C, relative humidity from 52 ± 9 to 96 ± 3%, and variable photosynthetic photon flux density (PPFD). To maintain vegetative growth, PPFD was initially supplemented for approximately 4 h per day with 50 W LED lamps, extending the photoperiod to about 16 h. Individuals were spaced 50 cm apart within rows and 75 cm between rows and were fertilized weekly with a commercial nutrient solution (DuGreen^®^) following the manufacturer’s recommendations.

When plants reached approximately 1 m tall (40 days after transplanting), the LED lamps were turned off to induce flowering, which occurred uniformly in each genotype. Until the onset of flowering, plants were irrigated to maintain substrate moisture close to FC. To this end, a water retention curve of the substrate was established [39] to determine both FC and the permanent wilting point, thereby enabling precise control of water application. After early flowering (46 days after transplanting), half of the plants of each genotype continued to be irrigated at FC, whereas the remaining plants were subjected to a stepwise reduction in water availability to 75, 50, 40, and 30% of FC, with each moisture level maintained for 5 d. During drought imposition, the soil surface of each pot was covered with plastic film to minimize evaporative water loss. Physiological measurements and sampling for subsequent biochemical analyses (see below) were performed in droughted plants on the fourth or fifth day after irrigation corresponding to 50 and 30% FC. Unless otherwise specified, measurements were taken on the youngest fully expanded leaves. The experiment lasted 75 days, at which point all plants were harvested for biomass analyses.

### 4.2. Leaf Water Potential and Pressure–Volume Curves

Leaf Ψ_w_ was determined at both predawn and midday using a Scholander-type pressure chamber (Model 1000; PMS Instrument Company, Albany, NY, USA). To prevent desiccation before measurement, leaves were excised and immediately sealed in zip-lock plastic bags containing a moistened paper towel. For pressure–volume curves, leaves were collected and sealed as described above. Samples were transferred to the laboratory (100 m away) and rehydrated to full turgor by immersing their petioles in water inside a humidity-saturated chamber for 12 h. Subsequently, leaf fresh mass (precision balance, 0.1 mg) and Ψ_w_ (Scholander chamber) were recorded periodically during a natural dehydration cycle until Ψ_w_ reached approximately –2.5 MPa. Leaves were then oven-dried at 60 °C until constant mass. Pressure–volume curves were constructed by plotting the relative water content against the inverse of Ψ_w_, following Tyree and Hammel [40]. From these curves, Ψ_s(100)_, Ψ_s(0)_, ε, *C*_(100)_, and *C*_(0)_ were determined, as described by Blackman and Brodribb [41].

### 4.3. Gas Exchange and Chlorophyll Fluorescence

Gas exchange (*A*, *g*_s_, *E*, and *C*_i_) and Chl *a* fluorescence were measured simultaneously using a portable photosynthesis system (LI-6400XT; LI-COR, Lincoln, NE, USA) fitted with an integrated fluorescence chamber (LI-6400-40). Gas-exchange measurements were recorded at 09h00−11h00 (solar time) at a PPFD of 1000 µmol m^−2^ s^−1^, 28 °C, and an ambient CO_2_ concentration of 430 µmol mol^−1^, following DaMatta et al. [42].

Leaf samples were first dark-adapted for 2 h, after which they were exposed to a weak modulated measuring beam (0.03 μmol m^−2^ s^−1^) to determine *F*_0_. Maximum fluorescence (*F*_m_) was then obtained by applying a saturating pulse of white light (8000 μmol photons m^−2^ s^−1^; 0.8 s). From these values, the maximum quantum efficiency of PSII photochemistry was calculated as *F*_v_/*F*_m_ = (*F*_m_ − *F*_0_)/*F*_m_. In light-acclimated tissues (1000 μmol m^−2^ s^−1^), steady-state fluorescence (*F*_s_) was measured immediately before another saturating pulse (8000 μmol m^−2^ s^−1^, 0.8 s), which yielded the maximum fluorescence under light (*F*_m_′). Following this, actinic illumination was switched off and a far-red beam (2 μmol m^−2^ s^−1^) was applied to obtain the minimal fluorescence in light-acclimated leaves (*F*_0_′). These parameters were then used to estimate several photosynthetic traits: the coefficient of photochemical quenching, *q*_L_ = [(*F*_m_′ − *F*s)/(*F*_m_′ − *F*_0_′)] × (*F*_0_′/*F*_s_), the non-photochemical quenching, NPQ = ((*F*_m_ *− F*_m_′)/*F*_m_′, and the effective quantum yield of PSII, *Φ*_PSII_ = (*F*_m_′ − *F*_s_)/*F*_m_′ [28]. ETR was subsequently derived as *Φ*_PSII_×PPFD×*f*α; where *f* represents the assumed 0.5 partitioning of excitation energy between PSI and PSII, and *α* corresponds to the leaf absorptance (set at 0.84) of photosynthetic tissues [43].

Nocturnal mitochondrial respiration (*R*_n_) was measured 2 h after darkness and used to estimate respiration in the light (*R*_d_) following Lloyd et al. [44]: *R*_d_ = (0.5 − 0.05 ln(PPFD)) × *R*_n_. The rate of photorespiration (*R*_p_) was then obtained according to Valentini et al. [45] using the equation *R*_p_ = 1/12 [*ETR* − 4(*A* + *R*_d_)], and the *R*_p_/*A*_G_ ratio was calculated as described by DaMatta et al. [42]. *V*_cmax_ was estimated with the one-point method [46], using tobacco RuBisCO kinetics (*K*_c_ = 8.6 µM; *K*_o_ = 226 µM) [47] together with simultaneous morning *A* and ETR, when both parameters typically reach their maximum. Finally, *V*_cmax_ values were standardized to 25 °C following the temperature correction procedure proposed by Sharkey et al. [48].

### 4.4. Biochemical and Cannabinoid Analyses

Leaf samples collected at midday were rapidly frozen in liquid nitrogen and stored at −80 °C until biochemical analyses. Tissues were subsequently lyophilized at −48 °C and ground with metallic beads in a Mini Bead Beater disruptor. For primary metabolite quantification, 10 mg aliquots were extracted in methanol [49]. The soluble fraction was used to determine photosynthetic pigments [50], hexoses and sucrose [51], total amino acids [52], total phenolics [53], and proline [54]; the insoluble fraction was analyzed for total proteins [55] and starch [49,56].

MDA was quantified from 30 mg of lyophilized tissue extracted in aqueous 80% (*v*/*v*) ethanol. The extract was split into two tubes: one received 20% (*w*/*v*) trichloroacetic acid (TCA) only, and the other received 20% (*w*/*v*) TCA containing 0.65% (*w*/*v*) thiobarbituric acid. MDA, used as a proxy for lipid peroxidation, was determined in the soluble fraction following Hodges et al. [57], with the corrections proposed by Landi [58]. For antioxidant enzymes, 20 mg aliquots were extracted as in Peixoto et al. [59] to determine activities of SOD (EC 1.15.1.1) [60], CAT (EC 1.11.1.6) [61], and POX (EC 1.11.1.7) [62]. Protein content of enzymatic extracts was measured by the Bradford assay [55] using bovine serum albumin as the standard, and specific enzyme activities were expressed on a protein basis.

The major cannabinoids (CBD, THC, and CBG) were quantified using a portable Fourier-transform near-infrared (FT-NIR) spectrometer (Valenveras Portable Lab, NeoSpectra, Barcelona, Spain) operating across the 1350–2500 nm range. Analyses were performed on oven-dried, ground inflorescences, following validated protocols for NIR-based cannabinoid profiling [63,64]. According to the manufacturer, the prediction models achieved coefficients of determination (R^2^) > 0.90 and errors comparable to those of standard HPLC assays. Total cannabinoid concentration was computed as the sum of CBD, THC, and CBG.

### 4.5. Growth Traits

At the end of the experiment, aboveground tissues were separated into leaves, stems, and inflorescences, which were oven-dried at 70 °C for 72 h to determine their dry mass. Senesced leaves—typically remaining attached to the stems but detaching easily when handled—were collected and their mass added to the total leaf dry mass. Plant height and stem diameter were measured with a ruler and a digital caliper, respectively. Roots were excluded from the analysis due to the difficulty of separating them from the substrate and the inevitable losses during washing. For each plant, two representative leaves were scanned prior to drying to estimate specific leaf area (SLA). Total leaf area was then calculated by multiplying SLA by total leaf biomass. The HI was computed as the ratio of inflorescence biomass to total aboveground biomass, following standard procedures used for field-grown crops.

### 4.6. Experimental Design and Statistical Analysis

The experiment followed a completely randomized factorial design with two genotypes and two water regimes. Each treatment consisted of eight replicates, with individual pots (one plant per pot) serving as the experimental unit. Data were first tested for normality (Shapiro–Wilk test) and homogeneity of variances (Levene’s test). As all assumptions were met, data were analyzed in R (RStudio 2024.12.1+536) using a two-way ANOVA at *p* ≤ 0.05. Effect sizes for genotype, water treatment, and their interaction were quantified as partial eta squared (ηp^2^), providing an estimate of the magnitude and biological relevance of each effect. Pairwise comparisons were then conducted (i) between genotypes within each soil moisture level (indicated by different letters) and (ii) between water treatments within each genotype (indicated by an asterisk when drought caused a significant effect).

## 5. Conclusions

In summary, the combined physiological, biochemical, and agronomic evidence demonstrates that drought responses in cannabis involve complex trade-offs between carbon assimilation, metabolite biosynthesis, and reproductive allocation, which differ markedly between genotypes.

Our results only partially supported the initial hypotheses. Although CBD plants were more vigorous, they did not sustain higher *A* and, under drought, experienced both stomatal and nonstomatal limitations that compromised their photosynthetic performance. In contrast, THC plants relied primarily on stomatal regulation to modulate *A* under water deficit. Despite genotypic contrasts in photoprotective responses, both genotypes converged toward similar levels of oxidative damage, indicating that photoprotection was not decisive for their divergence. At the agronomic level, THC plants maintained a higher HI under drought, greater baseline cannabinoid concentrations, and higher energetic value. In contrast, the sharper decline in cannabinoids observed in CBD plants was largely attributable to reduced photosynthesis and less efficient carbohydrate use, whereas in THC plants, the metabolic pathway itself was likely modified, as suggested by decreases in CBG pools. Overall, drought resilience in cannabis imposed at early flowering appears to depend less on hydraulic stability and more on safeguarding photosynthetic performance, sustaining secondary metabolism, and ensuring efficient biomass partitioning. These traits provide promising targets for breeding genotypes better adapted to water-limited environments.

## Figures and Tables

**Table 1:** Parameters from pressure–volume curves: osmotic potential at full turgor (Ψs_(100)_) and at the turgor loss point (Ψs_(0)_), bulk modulus of elasticity (Ɛ), and leaf capacitance at full turgor (*C*_(100)_) and at the turgor loss point (*C*_(0)_) in two cannabis genotypes grown under well-watered or drought conditions. Values are means ± SE (*n* = 5). Different letters indicate significant differences between genotypes within the same water regime; asterisks, when shown, indicate a drought effect within a genotype (Tukey’s test, *p* ≤ 0.05).

Parameters	CBD		THC	
	Control	Drought	Control	Drought
Ψs_(100)_ (MPa)	−0.73 ± 0.12 a	−0.62 ± 0.06 a	−1.08 ± 0.17 a	−0.69 ± 0.05 a
Ψs_(0)_ (MPa)	−1.23 ± 0.19 a	−1.13 ± 0.08 a	−1.50 ± 0.15 a	−1.08 ± 0.06 a
ε (MPa)	5.77 ± 1.12 a	4.49 ± 0.48 a	6.75 ± 1.21 a	5.82 ± 0.62 a
*C*_(100)_ (mol m^−2^ MPa^−1^)	0.09 ± 0.02 a	0.11 ± 0.01 a	0.09 ± 0.01 a	0.09 ± 0.01 a
*C*_(0)_ (mol m^−2^ MPa^−1^)	0.11 ± 0.02 b	0.11 ± 0.01 a	0.22 ± 0.03 a	0.11 ± 0.02 a *

**Table 2:** Maximum quantum efficiency of PSII photochemistry (*F*_v_/*F*_m_), minimum chlorophyll fluorescence (*F*_0_), coefficient of photochemical quenching (*q*_L_), and non- photochemical quenching (NPQ) in two cannabis genotypes grown under well-watered or drought conditions. Values are means ± SE (*n* = 8). Different letters indicate significant differences between genotypes within the same water regime; asterisks, when shown, indicate a drought effect within a genotype (Tukey’s test, *p* ≤ 0.05).

Parameters	CBD		THC	
	Control	Drought	Control	Drought
*F*_v_/*F*_m_	0.81 ± 0.01 a	0.64 ± 0.01 a *	0.83 ± 0.01 a	0.68 ± 0.02 a *
*F*_0_	626 ± 18 a	950 ± 72 a *	554 ± 19 a	922 ± 98 a *
*q*_L_	0.46 ± 0.02 a	0.19 ± 0.01 a *	0.44 ± 0.03 a	0.12 ± 0.07 a *
NPQ	0.96 ± 0.08 b	0.58 ± 0.07 b *	1.74 ± 0.28 a	1.15 ± 0.14 a *

**Table 3:** Activities of superoxide dismutase (SOD), catalase (CAT) and peroxidases (POX), and malondialdehyde (MDA) concentration in two cannabis genotypes grown under well-watered or drought conditions. Values are means ± SE (*n* = 5). Different letters indicate significant differences between genotypes within the same water regime; asterisks, when shown, indicate a drought effect within a genotype (Tukey’s test, *p* ≤ 0.05).

Parameters	CBD		THC	
	Control	Drought	Control	Drought
SOD (U min^−1^ mg^−1^ protein)	26.43 ± 1.9 a	16.73 ± 1.2 a *	19.85 ± 1.5 b	9.23 ± 0.6 b *
CAT (µmol H_2_O_2_ min^−1^ mg^−1^ protein)	17.5 ± 1.3 a	14.1 ± 0.9 a *	12.4 ± 0.7 b	8.7 ± 0.5 b *
POX (nmol purpurogalin min^−1^ mg^−1^ protein)	106 ± 12 a	31 ± 12 a *	41 ± 12 b	35 ± 10 a
MDA (µmol g^−1^ DW)	0.95 ± 0.09 a	1.24 ± 0.06 a *	1.16 ± 0.05 a	1.50 ± 0.09 a *

**Table 4:** Leaf concentrations (per dry mass) of chlorophylls, carotenoids, free amino acids, glucose, fructose, sucrose, starch, phenols and in two cannabis genotypes grown under well-watered or drought conditions. Values are means ± SE (*n* = 5). Different letters indicate significant differences between genotypes within the same water regime; asterisks, when shown, indicate a drought effect within a genotype (Tukey’s test, *p* ≤ 0.05).

Parameters	CBD		THC	
	Control	Drought	Control	Drought
Chlorophylls *a* + *b* (μg g^−1^)	2807 ± 133 a	1759 ± 131 b *	2813 ± 35 a	2320 ± 77 a *
Carotenoids (μg g^−1^)	735 ± 20 a	437 ± 33 a *	715 ± 9 a	522 ± 22 a *
Glucose (μmol g^−1^)	87 ± 10 a	55 ± 3 a *	84 ± 6 a	55 ± 4 a *
Fructose (μmol g^−1^)	30 ± 3 a	24 ± 3 a *	38 ± 5 a	29 ± 2 a *
Sucrose (μmol g^−1^)	93 ± 7 b	73 ± 3 b *	107 ± 2 a	82 ± 2 a *
Starch (mmol equiv. glucose g^−1^)	0.90 ± 0.09 a	0.73 ± 0.06 a *	0.60 ± 0.07 b	0.42 ± 0.07 b *
Amino acids (μmol g^−1^)	43 ± 2 a	30 ± 2 a *	44 ± 1 a	32 ± 2 a *
Phenolics (mg g^−1^)	113 ± 3 a	102 ± 6 a	89 ± 2 b	91 ± 4 b
Proline (μmol g^−1^)	9.81 ± 0.5 a	8.09 ± 0.5 a *	10.05 ± 1.0 a	7.72 ± 0.5 a *

## Data Availability

The original contributions presented in this study are included in the article/supplementary material. Further inquiries can be directed to the corresponding author.

## Supplementary Materials

The following supporting information can be downloaded at: https://www.mdpi.com/article/10.3390/plants14243840/s1.
