# Supplementary material for: Genotypic Variation in Photosynthesis and Biomass Partitioning Underlies Agronomic Performance and Cannabinoid Profile in Cannabis sativa Under Drought
Source: Plants (Basel). 2025 Dec 17;14(24):3840. doi: 10.3390/plants14243840 (PMC12736467; doi:10.3390/plants14243840)
Supplement: Supplementary file 1 [file plants-14-03840-s001.zip › plants-4011504-supplementary.pdf]

Table S1. Results of two-way ANOVA and effect size analysis showing the effects of genotype (G), water availability (W), and their interaction (G × W) on plant water relations, gas exchange, biochemical, metabolic, growth, and cannabinoid-related traits. For each variable, *F* values and significance levels are reported together with partial eta squared (ηp²), which quantifies the proportion of variance explained by each factor and provides an estimate of effect magnitude and biological relevance. Significant effects are indicated at $P \leq 0.05$ (*), $P \leq 0.01$ (**), and $P \leq 0.001$ (***). Abbreviations are defined in the main text.

Parameter	*F* (Gen)	η^2p (Gen)	F (Water)	η^2p (Water)	F (G x W)	η^2p (G x W)
$\Psi_{pd}50$	1.3	0.055	5.0*	0.185	3.3	0.131
$\Psi_{md}50$	5.3*	0.180	11**	0.315	1.8	0.069
$\Psi_{pd}30$	1.3	0.074	15**	0.489	0.167	0.010
$\Psi_{md}30$	2.8	0.123	97***	0.830	0.029	0.001
$\Psi S_{(100)}$	1.1	0.062	1.2	0.069	0.463	0.028
$\Psi S_{(0)}$	0.418	0.025	0.796	0.047	0.768	0.046
ε	2.6	0.137	0.375	0.023	0.209	0.013
$C_{(100)}$	1.7	0.094	0.141	0.008	0.032	0.002
$C_{(0)}$	25***	0.613	21***	0.574	20***	0.568
*A*50	3.7	0.123	41***	0.616	0.064	0.002
*A*30	27***	0.511	110***	0.810	4.9*	0.158
g_s50	2.2	0.079	146***	0.850	0.880	0.033
g_s30	0.027	0.001	71***	0.746	1.4	0.056
*E*50	0.246	0.009	152***	0.854	0.383	0.015
*E*30	4.0	0.134	69***	0.726	1.4	0.051
C_i50	0.526	0.020	84***	0.764	1.2	0.044
C_i30	0.032	0.001	41***	0.617	0.0004	0.0002
ETR50	2.0	0.078	0.520	0.022	0.423	0.018
ETR30	1.0	0.044	1.6	0.066	0.425	0.019
$V_{cmax}50$	2.2	0.127	1.4	0.087	0.019	0.001
$V_{cmax}30$	4.0*	0.205	4.3*	0.232	3.8	0.106
ETR/*A*50	0.165	0.008	31.2***	0.609	0.013	0.0007
ETR/*A*30	9.0**	0.322	18.3***	0.490	7.2*	0.274
R_P/A_G50	0.305	0.013	23***	0.499	0.035	0.002
R_P/A_G30	47***	0.703	71***	0.779	17***	0.463
F_v/F_m	1.3	0.048	21***	0.450	0.303	0.012
F_0	0.808	0.030	35***	0.576	0.224	0.009
q_L	0.290	0.024	39***	0.768	0.024	0.002
NPQ	16**	0.568	11**	0.480	0.256	0.021
SOD	34***	0.678	49***	0.755	0.084	0.005
CAT	34***	0.682	21***	0.571	0.157	0.010
POX	2.8	0.149	8.7**	0.352	5.0*	0.238
MDA	3.9	0.198	14**	0.477	0.426	0.026
Chl *a+b*	7.6*	0.321	56***	0.778	7.2*	0.311
Carotenoids	1.9	0.108	114***	0.877	2.2	0.124
Glucose	0.045	0.003	48***	0.788	0.888	0.064
Fructose	4.3	0.262	14**	0.535	0.312	0.025
Sucrose	10**	0.417	35***	0.713	0.103	0.007

Starch	19***	0.610	13**	0.525	0.030	0.002
Amino acids	1.0	0.060	50***	0.758	0.053	0.003
Phenols	17***	0.512	1.3	0.076	2.5	0.135
Proline	0.651	0.039	14**	0.475	1.8	0.101
CBD	939***	0.978	13**	0.388	13**	0.375
THC	927***	0.987	16**	0.575	15**	0.562
CBG	490***	0.959	6.5*	0.235	29***	0.582
Total cannabinoids	327***	0.942	8.5**	0.298	4.6*	0.131
Total biomass	55***	0.680	4.9*	0.158	6.7*	0.205
Stem biomass	26***	0.503	0.166	0.006	0.095	0.004
Leaf biomass	4.8*	0.156	0.090	0.003	0.084	0.003
Leaf area	0.795	0.033	6.7*	0.225	0.308	0.013
Flower biomass	39***	0.599	58***	0.689	50***	0.657
Harvest index	7.5*	0.223	5.2*	0.166	4.5*	0.152
